# Effect Estimate of Time-varying Social Support and Trust on the Physical and Mental Health of Mothers at 2.5 Years Postpartum: The Japan Environment and Children’s Study (JECS)

**DOI:** 10.2188/jea.JE20210134

**Published:** 2023-04-05

**Authors:** Kenta Matsumura, Ryoko Morozumi, Kei Hamazaki, Akiko Tsuchida, Hidekuni Inadera

**Affiliations:** 1 Toyama Regional Center for JECS, University of Toyama, Toyama, Japan; 2 Department of Public Health, Faculty of Medicine, University of Toyama, Toyama, Japan; 3 Faculty of Social Sciences, University of Toyama, Toyama, Japan; 4 Department of Public Health, Gunma University Graduate School of Medicine, Gunma, Japan

**Keywords:** SF-8, emotional support, inverse probability of treatment weighting, causal association, pregnant women

## Abstract

**Background:**

Many epidemiological studies have reported the association between various social factors and health status in mothers during and after pregnancy. However, little is known about their joint and longitudinal impact. We examined the association of lack of social support and trust during pregnancy and at 2.5 years postpartum with health status in mothers.

**Methods:**

To adequately address time-varying exposure, marginal structural models were fitted to a pseudopopulation constructed using inverse probability weighting. The model included records of 90,071 mothers participating in the Japan Environment and Children’s Study. Social support and trust were measured using a 9-item questionnaire (Q1–9). Mental and physical health were measured using Mental and Physical Component Summary scores from the 8-item Short-Form Health Survey.

**Results:**

For the Mental Component Summary, the magnitude of the effect estimate was largest when participants lacked close friends/neighbors (Q4) at only 2.5 years postpartum (effect estimate, −6.23), followed by a lack in emotional support (Q2) at the same time point (effect estimate, −4.94). For the Physical Component Summary, effect estimates were negligible. The magnitude of the effect estimates of lack of social support and trust tended to be larger when there was a lack at only 2.5 years postpartum than at both time points.

**Conclusion:**

These findings suggest that, after childbirth, a loss in social support, particularly in an emotional aspect, carries high risk, especially for mental health. Our results highlight the importance of supporting mothers for more than a few years after pregnancy.

## INTRODUCTION

Women have heightened vulnerability to health problems during the perinatal and postpartum period. Examples include fatigue and pain,^1^ depressive symptoms,^2^^,^^3^ anxiety,^4^^–^^6^ and bonding failure.^7^ Recent studies have revealed that such health problems can continue for several years after childbirth. For example, the reported prevalence of chronic pain due to childbirth is 6.1% at 2.3 years postpartum^8^ and 9.3% from 1 to 5 years,^9^ and the prevalence of depression at 2 years postpartum is 13%.^10^ Such chronic health problems not only reduce quality of life,^8^^,^^11^ but also are associated with behavioral and psychiatric problems in children.^12^^–^^14^ Therefore, studies examining postpartum mothers should pay more attention to these problems over a prolonged period of time.

Various factors have been linked to the health problems associated with childbirth. One such factor is social support. Previous studies have found that social support was a protective factor not only in the early months, but also for several years postpartum^10^^,^^15^ and that it had a relatively large effect size^16^^,^^17^ and was associated with both physical and mental health.^18^^,^^19^ However, what remains unclear is the impact of social support at different time points on prolonged health problems. Although studies have investigated various aspects of social support, such as those addressing prolonged health problems,^8^^,^^9^^,^^20^^–^^24^ measuring social support twice or more,^15^^,^^23^^,^^25^^–^^28^ or directly comparing their joint effect on mothers’ health,^25^ no study has simultaneously examined all of these properties. Given that social support can change over time and could be targeted in an intervention, the impact of social support at different time points on prolonged health problems is worth examining.

We previously examined the causal association of social support and trust with self-rated health, measured using the 8-Item Short-Form Health Survey (SF-8),^29^^–^^31^ in expectant mothers in Japan^19^ and revealed a favorable effect specifically in the mental component. Continuing on from this work,^19^ we examined the association of social support and trust during pregnancy and at 2.5 years postpartum with mental and physical health in mothers at 2.5 years postpartum in the present study. In particular, we compared mothers who lacked social support and trust at either of the two time points or at both of them with those who did not lack them at any time point. Our aim was to determine the impact of social support and trust at different time points and their combinations on prolonged health problems. To adequately address time-varying exposure and confounders as well as loss to follow-up,^32^^,^^33^ we applied marginal structural models (MSMs) to a pseudopopulation that was created using inverse probability (IP) weightings and then made effect estimates.

## METHODS

### Study population

**Figure 1.**
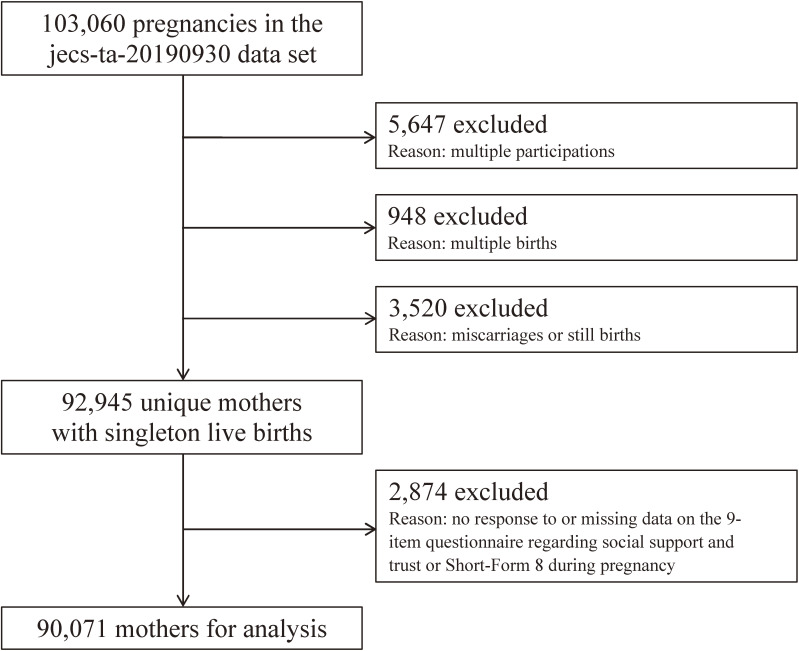
Participant flow chart

The study data were obtained from mothers participating in the Japan Environment and Children’s Study (JECS). The JECS is an ongoing nationwide government-funded birth cohort study focusing on various environmental factors and child health and development. The design of the JECS has been reported in detail elsewhere.^34^^–^^36^ Briefly, the participants were enrolled from 15 regional centers in Japan (including both rural and urban locations from the subarctic northern island of Hokkaido to the subtropical southern island of Okinawa) via face-to-face recruitment at the first trimester between January 2011 and March 2014. Follow-ups were conducted at the second/third trimester, at childbirth, and at 1 month postpartum during scheduled in-hospital checkups. Subsequent follow-ups were conducted at 6, 12, 18, 24, 30, and 36 months postpartum via mailed letters. The present study analyzed the jecs-ta-20190930 data set, which was released in October 2019. This data set includes data on 103,060 pregnancies up to 36 months postpartum, from which we excluded 5,647 multiple participations (the second or third registration of the same mother), 948 multiple births (twins or more), 3,520 miscarriages/still births, and 2,874 records completely missing data on the 9-item questionnaire regarding social support and trust (described below) or the SF-8 during pregnancy, leaving 90,071 mothers for the final analysis (Figure 1).

The JECS protocol was reviewed and approved by the Ministry of the Environment’s Institutional Review Board on Epidemiological Studies and the Ethics Committees of all participating institutions. Written informed consent was provided by all participants.

### Measures

#### Outcomes

Mothers’ health was measured twice—during pregnancy and at 2.5 years after childbirth—using the SF-8.^29^^–^^31^ The SF-8 comprises eight items assessing general health, physical functioning, role-physical, bodily pain, vitality, social functioning, mental health, and role-emotional. Mental Component Summary (MCS) and Physical Component Summary (PCS) scores derived from the SF-8 were used in this study. The MCS and PCS scores are continuous and standardized to a national mean of 50 and standard deviation (SD) of 10, with higher scores indicating better health status. The SF-8 yields scores that are highly predictable and directly comparable to those of the MOS 36-item Short-Form Health Survey.^29^^–^^31^

#### Exposures

Social support and trust at the second/third trimester and at 2.5 years after childbirth were assessed using a nine-item questionnaire.^18^ Questionnaire items and response categories are listed in Table 1 and comprise questions on social support (4 items), neighborhood trust (3 items), and generalized trust (2 items). Social support items were derived from the ENRICHD Social Support Inventory^37^ with some modifications and included items Q1–4. Neighborhood trust items were derived from the questionnaire used in the Project on Human Development in Chicago Neighborhoods^38^ with some modifications and included items Q5–7. Generalized trust items were derived from the Trust in People Scale^39^ with some modifications and included items Q8–9.

**Table 1.** The 9-item social support and trust questionnaire

Item		Percentage of cases	

		at 2^nd^/3^rd^ trimester	at 2.5 years postpartum
Social support			
Q1	Is there someone available to you who shows you love and affection?	4.3	2.7
	(1 = None of the time, 2 = A little of the time, 3 = Some of the time, 4 = Most of the time, 5 = All of the time)		
Q2	Is there someone whom you can count on to provide you with emotional support (talking over problems or helping you make a difficult decision)?	2.2	1.7
	(1 = None of the time, 2 = A little of the time, 3 = Some of the time, 4 = Most of the time, 5 = All of the time)		
Q3	How often do you have as much contact as you would like with someone you feel close to: someone in whom you can trust and confide?	1.6	2.6
	(1 = None of the time, 2 = A little of the time, 3 = Some of the time, 4 = Most of the time, 5 = All of the time)		
Q4	Number of friends/neighbors to whom you can talk casually about your concern	1.0	1.4
	(1 = 0, 2 = 1 or 2, 3 = 3 or more)^a^		
Neighborhood trust			
Q5	Neighbors trust each other	20.5	14.4
	(1 = Agree, 2 = Somewhat agree, 3 = Somewhat disagree, 4 = Disagree)^b^		
Q6	Neighbors help each other	19.9	14.1
	(1 = Agree, 2 = Somewhat agree, 3 = Somewhat disagree, 4 = Disagree)^b^		
Q7	Do you think your neighborhood is safe?	8.4	4.7
	(1 = Yes, 2 = Don’t know, 3 = No)^b^		
Generalized trust			
Q8	Would you say that most people can be trusted?	3.4	5.2
	(1–9 = Most people can be trusted–You can’t be too careful)		
Q9	Would you say that most of the time people try to be helpful, or that they are mostly just looking out for themselves?	1.9	1.9
	(1–9 = Helpful–Look out for self)		

^a^Choices at 2.5 years postpartum were (1 = 0, 2 = 1, 2, or 3, 3 = 4 or more).

^b^Choices at 2.5 years postpartum were (1 = Agree, 2 = Somewhat agree, 3 = Somewhat disagree, 4 = Disagree, 5 = Don’t know).

See text for details.

Because our previous study^19^ revealed a salient reduction in self-rated health in the lowest response category in each item (“None of the time” response to Q1–Q3, “None” response to Q4, “Disagree” response to Q5–Q7, “You cannot be too careful” response to Q8, and “Look out for self” response to Q9) and because, even though the response categories in Q4–7 during pregnancy and at 2.5 years postpartum were slightly different, at least the lowest response categories during pregnancy and at 2.5 years postpartum were the same for all items, in the present study we re-defined each of these answers as a lack of social support or trust—a binary variable—and used this as an exposure variable.

#### Covariates

Based on the previous study,^19^ we selected potential baseline confounders, defined as variables before and/or during pregnancy with a theoretical impact on both exposure and outcome, as well as basic anthropometric and socioeconomic variables. These variables included maternal age, pre-pregnancy body mass index, parity, marital status, highest education level, employment status, annual household income, smoking status, alcohol intake, history of any physical disease, history of major psychiatric disease, pregnancy complication, intimate partner violence, negative attitude toward pregnancy, and stressful events.

In addition, we selected post-childbirth covariates, defined as variables that appeared after baseline or time-dependent variables that can be affected by previous variables. These included the child’s sex, cesarean delivery, preterm birth, presence of a major congenital anomaly,^40^ repeat pregnancy, divorced or widowed status, job loss, annual household income, smoking status, alcohol intake, disease or injury, stressful events, children’s experience of nursery, and moving houses.

The variables were categorized according to standard medical practice, common practice in Japan, and/or the results of previous studies.^41^^–^^43^ The categorization is shown in Table 1.

### Statistical analysis

#### Marginal structural models

**Figure 2.**
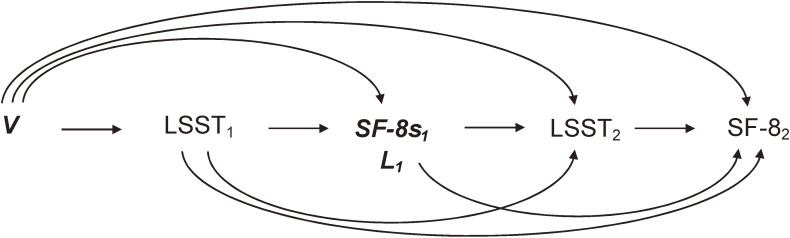
Causal diagram of the association of a time-varying Lack of Social Support and Trust (LSST) at the second/third trimester (LSST_1_) and at 2.5 years postpartum (LSST_2_) with the SF-8 score at 2.5 years postpartum (SF-8_2_). LSST = 1 if women had a lack of social support and trust and 0 otherwise. ***SF*-8*s*_1_** represents the SF-8 scores (MCS and PCS) at the second/third trimester. ***V*** represents baseline confounders: maternal age, pre-pregnancy body mass index, parity, marital status, highest education level, employment status, annual household income, smoking status, alcohol intake, history of any physical disease, history of major psychiatric disease, pregnancy complication, intimate partner violence, negative attitude toward pregnancy, and stressful events. ***L*_1_** represents time-varying covariates or covariates that appeared after baseline, including child’s sex, cesarean delivery, preterm birth, major congenital anomaly of the baby, repeat pregnancy, divorced or widowed status, job loss, annual household income, smoking status, alcohol intake, disease or injury, stressful event, children’s experience of nursery, and moving house.

MSMs were fitted to the pseudopopulation using the IP-weighted least squares approach to appropriately manage time-varying exposures and covariates and censoring due to loss to follow-up. A directed acyclic graph of this study is shown in Figure 2. To closely estimate the effects of the lack of different types of social support or trust on different health statuses, we fitted 18 sets of MSMs (9 types [Q1–9] × 2 scores [MCS or PCS]). The MSM took the following form:

$$\begin{array}{rl} E[{\text{Score}}_{2}{}^{\text{LSST}1,\text{LSST}2}] & =\beta_{0}+\beta_{1}{LSST}_{1}+\beta_{2}{LSST}_{2}+\beta_{3}{LSST}_{1}\\ & \;\times {LSST}_{2} \end{array}$$

where *Score*_2_*^LSST^*^1,^*^LSST^*^2^ were counterfactual outcomes for if mothers had all undergone a combination of interventions to set the type at the second/third trimester and at 2.5 years after childbirth, contrary to fact, to LSST_1_ and LSST_2_, respectively (LSST = 1 if women had a lack of social support or trust, otherwise 0). Here, the effect estimates of a lack of social support or trust at only the second/third trimester, at only 2.5 years after childbirth, and at both time points are expressed as β_1_, β_2_, and β_1_ + β_2_ + β_3_, respectively. β_0_ is a counterfactual score for no lack of social support or trust at any time point. β_0_ serves as a reference intercept value.

#### Inverse probability of treatment weights

We used the stabilized IP of treatment weights (*SW_A_*) to construct a pseudopopulation:

$$\begin{array}{rl} {SW}_{A} & =Pr[{LSST}_{1}]/Pr[{LSST}_{1}|V]\\ & \;\times Pr[{LSST}_{2}|{LSST}_{1}]/Pr[{LSST}_{2}|{LSST}_{1},{\text{Scores}}_{1},V,L_{1}] \end{array}$$

where *V* and *L*_1_ represent the baseline potential confounders and post-childbirth covariates mentioned above, respectively, and *Scores*_1_ represents the MCS and PCS scores at the second/third trimester.

#### Inverse probability of censoring weights

We used stabilized IP weights for censoring (*SW_C_*) to avoid the selection bias involved in censoring due to loss to follow-up:

$${SW}_{C}=Pr[C=0|{LSST}_{1}]/Pr[C=0|{LSST}_{1},{\text{Scores}}_{1},V]$$

Here, *C* = 1 if a participant was lost to follow-up at 2.5 years after childbirth and *C* = 0 otherwise.

#### Final inverse probability weights

The final stabilized IP weight of each participant (*SW_A_*_,_*_C_*) was calculated as

$${SW}_{A,C}={SW}_{A}\times {SW}_{C}$$

#### Sensitivity analysis

To evaluate unmeasured confounding, we calculated the *E*-value,^44^^,^^45^ which is the minimum association required to completely cancel out the observed association. Because the outcomes were continuous values, we first calculated Cohen’s *d*^46^ and then transformed the value into the risk ratio (RR) using the following equation: RR ≈ exp(0.91 × *d*).^44^ We then further conducted an inverse conversion to yield *E*-values in Cohen’s *d*.

#### Additional analysis

To check for multicollinearity, we calculated generalized variance inflation factors.

All analyses were performed using SAS software (version 9.4; SAS Institute Inc., Cary, NC, USA) or R 4.0.0 (R Foundation for Statistical Computing, Vienna, Austria).

### Missing data

The effective response rate at 2.5 years after childbirth was 84.6% (76,163/90,071). Dropouts were treated as censored with SW_C_, as mentioned above.

Of the 90,071 mothers at baseline, the missing data rate was ≤1% for most baseline covariates, with the exceptions of parity (2.49%, *n* = 2,240) and annual household income (6.87%, *n* = 6,190). Of the 76,163 mothers at 2.5 years after childbirth, the missing data rate was ≤2.2% for most covariates, with the exception of repeat pregnancy (4.04%, *n* = 3,079) and annual household income (6.08%, *n* = 4,632).

We conducted imputation using chained equations^47^ to obtain 10 imputed data sets. When conducting multiple imputations, we also included auxiliary variables related to possible confounders to prevent violation of the assumption of missing at random. The results were combined using standard rules.^48^

## RESULTS

A total of 90,071 mothers were analyzed, of whom 74.0% were younger than 35 years of age, 73.3% had a pre-pregnancy body mass index of 18.5 to <25, and 42.6% were primiparous. Details of the participants’ characteristics are presented in Table 2. Mean MCS scores at the second/third trimester and at 2.5 years after childbirth were 49.03 (SD, 6.24) and 47.78 (SD, 6.56), respectively. Mean PCS scores at these time points were 45.71 (SD, 6.20) and 49.25 (SD, 6.37), respectively. The percentages of cases with a lack of social support or trust at the second/third trimester or at 2.5 years postpartum are summarized in Table 2. Compared with the mothers included (*n* = 90,071), those who were excluded from the analysis (*n* = 2,874) tended to experience pregnancy complications (Cramer’s *V* = 0.078), be younger (Cramer’s *V* = 0.032), be not married (Cramer’s *V* = 0.028), have no history of physical disease (Cramer’s *V* = 0.028), and have a lower education level (Cramer’s *V* = 0.026).

**Table 2.** Participants’ characteristics

Variable	Category	*n*	(%)
During pregnancy			

Age, years	<25	9,395	(10.4)
	25 to <30	25,521	(28.3)
	30 to <35	31,700	(35.2)
	≥35	23,440	(26.0)
Pre-pregnant body mass index, kg/m^2^	<18.5	14,600	(16.2)
	18.5 to <25	65,991	(73.3)
	≥25	9,425	(10.5)
Parity	Primiparous	37,429	(42.6)
	Multiparous	50,402	(57.4)
Marital status	Married	85,236	(95.5)
	Single	3,228	(3.6)
	Divorced or widowed	768	(0.9)
Highest education level of mother, years	≤12	32,359	(36.0)
	>12 to <16	37,821	(42.1)
	≥16	19,602	(21.8)
Employed	No	41,050	(45.9)
	Yes	48,352	(54.1)
Annual household income, million yen	<4	33,608	(40.1)
	4 to <6	27,710	(33.0)
	≥6	22,563	(26.9)
Smoking status	Never	51,727	(57.9)
	Former	33,577	(37.6)
	Current	4,048	(4.5)
Alcohol intake	Never	29,833	(33.4)
	Former	57,051	(63.8)
	Current	2,488	(2.8)
History of any physical disease	No	15,535	(17.3)
	Yes	74,087	(82.7)
History of psychiatric disease (anxiety, depression, autonomic dysregulation, and schizophrenia)	No	76,796	(85.7)
	Yes	12,826	(14.3)
Pregnancy complication	No	73,782	(82.1)
	Yes	16,059	(17.9)
Intimate partner violence	No	77,343	(86.0)
	Yes	12,595	(14.0)
Negative attitude toward pregnancy	No	82,631	(92.6)
	Yes	6,633	(7.4)
Stressful events	No	50,439	(56.4)
	Yes	38,997	(43.6)

Up to 2.5 years			

Cesarean delivery	No	72,776	(81.2)
	Yes	16,840	(18.8)
Birth weeks	≥37	85,781	(95.5)
	34 to <37	3,274	(3.6)
	<34	786	(0.9)
Child’s sex	Male	46,187	(51.3)
	Female	43,874	(48.7)
Major baby anomaly	No	87,931	(97.6)
	Yes	2,140	(2.4)
Repeat pregnancy	No	49,629	(67.9)
	Yes	23,455	(32.1)
Divorced or widowed	No	72,431	(97.1)
	Yes	2,183	(2.9)
Job loss	No	72,196	(96.9)
	Yes	2,278	(3.1)
Annual household income^a^, million yen	<4	23,648	(33.1)
	4 to <6	25,327	(35.4)
	≥6	22,556	(31.5)
Smoking status^b^	No	73,391	(92.5)
	Current	5,917	(7.5)
Alcohol intake^b^	No	55,022	(69.1)
	Current	24,600	(30.9)
Disease or injured	No	63,096	(83.6)
	Yes	12,377	(16.4)
Stressful events	No	28,475	(35.8)
	Yes	51,104	(64.2)
Nursery	No	38,915	(45.1)
	Yes	47,392	(54.9)
Move from area of recruitment center^a^	No	71,641	(95.4)
	Yes	3,432	(4.6)

^a^Measured at 3 years postpartum.

^b^Measured at 1.5 years postpartum.

Mean values of SW_A_, SW_C_, and SW_A,C_ ranged from 0.999 to 1.009 and the SDs ranged from 0.100 to 0.313 (eTable 1). Effect estimates for MCS and PCS scores according to the type of lack of social support and trust (Q1–9) are presented in Table 3. For MCS scores, the magnitude of the effect estimate was the largest for a lack in Q4 at only 2.5 years after childbirth (effect estimate, −6.23), followed in order by a lack in Q2 at only 2.5 years (effect estimate, −4.94) and a lack in Q2 at both the second/third trimester and 2.5 years after childbirth (effect estimate, −4.85). The value was the smallest for a lack in Q1 at only the second/third trimester (effect estimate, −0.22). All effect estimates had negative values, and all of the 95% confidence intervals (CIs) except two did not cross zero, which indicates the overall adverse effects of a lack on MCS scores. All effect estimates of interaction had positive values and the 95% CIs for Q3–8 did not cross zero. Mean effect estimates across Q1–9 at the second/third trimester and at 2.5 years postpartum were −1.23 and −3.15, respectively.

**Table 3.** Effect estimates for MCS and PCS scores according to the type of a lack of social support and trust

	Time point of lack		Item									Mean
	
	at 2^nd^/3^rd^ trimester	at 2.5 years postpartum	Social support				Neighborhood trust			Generalized trust		
		
			Q1	Q2	Q3	Q4	Q5	Q6	Q7	Q8	Q9	
MCS score												
β_0_ (Intercept)	No	No	47.75	47.78	47.83	47.79	48.07	48.03	47.86	47.85	47.76	47.86
Effect estimate			(0.05)	(0.05)	(0.05)	(0.05)	(0.06)	(0.06)	(0.05)	(0.05)	(0.05)	
β_1_	Yes	No	−0.22	−0.28	−1.96	−2.43	−0.77	−0.77	−1.19	−1.68	−1.73	−1.23
			(0.29)	(0.38)	(0.62)	(0.79)	(0.16)	(0.16)	(0.23)	(0.45)	(0.68)	
β_2_	No	Yes	−1.62	−4.94	−4.55	−6.23	−1.84	−1.68	−1.88	−1.97	−3.64	−3.15
			(0.43)	(0.58)	(0.44)	(0.66)	(0.19)	(0.19)	(0.33)	(0.32)	(0.67)	
β_1_ + β_2_ + β_3_	Yes	Yes	−0.97	−4.85	−4.10	−3.98	−1.91	−1.74	−1.92	−2.69	−3.82	−2.89
			(0.92)	(1.79)	(1.21)	(1.66)	(0.23)	(0.23)	(0.45)	(0.67)	(1.62)	
Interaction alone												
β_3_			0.86	0.37	2.41	4.68	0.70	0.71	1.15	0.96	1.56	1.49
			(1.05)	(1.92)	(1.43)	(1.96)	(0.33)	(0.33)	(0.60)	(0.87)	(1.88)	

PCS score												
β_0_ (Intercept)	No	No	49.22	49.23	49.23	49.23	49.32	49.31	49.31	49.24	49.22	49.26
Effect estimate			(0.05)	(0.05)	(0.05)	(0.05)	(0.05)	(0.05)	(0.05)	(0.05)	(0.05)	
β_1_	Yes	No	−0.17	−0.29	−0.62	−1.22	−0.33	−0.34	−0.83	−0.62	−0.98	−0.60
			(0.27)	(0.36)	(0.53)	(0.72)	(0.15)	(0.15)	(0.21)	(0.41)	(0.58)	
β_2_	No	Yes	0.08	0.14	−0.33	−0.17	−0.29	−0.34	−0.86	−0.11	−0.05	−0.22
			(0.39)	(0.47)	(0.40)	(0.55)	(0.17)	(0.18)	(0.31)	(0.27)	(0.51)	
β_1_ + β_2_ + β_3_	Yes	Yes	0.17	0.13	−1.09	−1.61	−0.69	−0.51	−0.83	−0.32	0.33	−0.49
			(0.80)	(1.22)	(1.52)	(1.69)	(0.22)	(0.22)	(0.41)	(0.63)	(1.21)	
Interaction alone												
β_3_			0.26	0.28	−0.14	−0.22	−0.07	0.18	0.86	0.41	1.36	0.33
			(0.93)	(1.36)	(1.66)	(1.91)	(0.31)	(0.31)	(0.55)	(0.80)	(1.43)	

MCS, Mental Component Summary derived from the SF-8PCS; Physical Component Summary derived from the SF-8; SF-8, 8-item Short-Form Health Survey.

Values are expressed as the mean (1.96 × SE).

For PCS scores, the magnitude of the effect estimate was the largest for a lack in Q4 at both the second/third trimester and 2.5 years after childbirth (effect estimate, −1.61), followed by a lack in Q4 at only the second/third trimester (effect estimate, −1.22). The largest value was for a lack in Q9 at both time points (effect estimate, 0.33). A total of 22 out of the 27 effect estimates had negative values and the 95% CIs did not cross zero in about half (13 of 27). No positive effect estimates had 95% CIs that did not cross zero. These results indicate weak adverse effects of a lack on PCS scores. The 95% CI did not cross zero for only one effect estimate of interaction (Q7). Mean effect estimates across Q1–9 at the second/third trimester and at 2.5 years postpartum were −0.60 and −0.22, respectively.

The *E*-values in Cohen’s *d* corresponding to effect estimates of 6.23 and 4.94 were 1.18 and 1.01, respectively, suggesting that relatively strong unmeasured covariates would be necessary to cancel out the estimated effects.

All generalized variance inflation factors were below 2.11, so no multicollinearity was detected among any of the covariates.

## DISCUSSION

As an extension of our previous study,^19^ we used JECS data to calculate the effect estimates of a time-varying lack of various types of social support or trust on the MCS and PCS scores of the SF-8 in mothers using MSMs fitted to a pseudopopulation created by IP weightings. The main findings of this study are as follows: (1) the effect estimate of a lack was highest for Q4 in the MCS, which was consistent with the results of our previous study^19^ and was about a 6.2-point decrease; (2) the second largest effect was observed for Q2 in the MCS and was about a 4.9-point decrease; (3) the mean effect estimates 2.5 years after childbirth (3.15-point decrease) were about 2.6 times as large as those at the second/third trimester (1.23-point decrease) in the MCS; (4) due to relatively large interactions, counterfactual MCS scores based on a lack at both time points were not always lower than those at only 2.5 years after childbirth; and (5) the mean effect estimates of a lack on physical health (ie, 0.60- and 0.22-point decrease at each the time point, respectively) were much smaller than those in the MCS (ie, 3.15- and 1.23-pont decreases, respectively). Taken together, our findings suggest that social support and trust, particularly regarding the emotional support aspect, are needed 2.5 years after childbirth to maintain mental health, but is largely not needed to maintain physical health, and that this need is especially strong when there is a lack during pregnancy. These findings suggest that a loss of social support after childbirth, particularly in the emotional support aspect, carries high risk for mental health.

The effects of a lack of social support or trust on MCS scores varied according to type. Overall, the magnitude of the effect estimate was largest in the “social support” subcategory (comprising Q1–4), followed in order by “generalized trust” (comprising Q8–9) and “neighborhood trust” (comprising Q5–7). However, individually, this was not always true: the effect estimate 2.5 years after childbirth was −1.62 in Q1 on emotional support, whereas it was −3.64 in Q9 on general trust. Given the common aspects in items with a large magnitude of the effect estimate (namely, Q4, Q2, Q3, and Q9), this result might be related to the likelihood of whether mothers receive concrete support from others. Mothers may experience a challenging time at the 2.5-year postpartum follow-up for several possible reasons, including being in the middle of the terrible twos; continued sleep disturbance or pain after delivery; the combined burden of a job and housekeeping work on mothers; unpredictable life events, such as job loss and bereavement; and deterioration of the maternal relationship. As such, the mental health of mothers might be improved with practical advice concerning childrearing, work, housekeeping, relaxing, and family relations. This might also be why the overall effect estimates 2.5 years after childbirth were larger than those at the second/third trimester in the MCS. Thus, mothers need help at this stage and not only during pregnancy.

The β_3_ interaction was the largest in Q4 (4.68), which resulted in a lower counterfactual MCS score with a lack at only 2.5 years postpartum compared with that at both time points. Interestingly, such interactions were also detected for Q3–8 of the MCS, but for only Q7 of the PCS. All interactions had positive values, which means that a lack of social support or trust at both time points tended to cancel each other out. Although continuous adverse circumstances might make mothers more resilient, such an adverse situation should obviously be avoided if possible—the MCS score when there was a lack at both time points never exceeded that when it was lacking at either time point. From a different perspective, the loss of social support or trust after childbirth would carry higher risk than their continuous lack. We can regard this interaction as a kind of gain-loss effect.^49^ Incidentally, such recovering interactions were not observed in a previous study that examined antepartum depression.^25^ However, that study conducted two measurements during pregnancy, whereas our study straddled childbirth. Spontaneous recovery likely requires situational changes or a longer time span. In any case, a system to assess the health status of mothers would be necessary, such as one that takes advantage of the routine medical checkup of their toddlers. Because periodic medical checkups become less frequent after childbirth, it is important for caregivers to determine which mothers are at risk.

Because MCS and PCS are standardized scores, these scales can be directly compared. The lower PCS values observed during pregnancy are likely due to negative effects of pregnancy on physical functioning. In contrast, the lower MCS scores 2.5 years after childbirth are likely because this is a challenging period, as mentioned above. Given that the PCS score at the second/third trimester was 45.71 (and thus a 4.3-point decrease from the mean) but that the MCS score 2.5 years after childbirth was 47.78 (and thus a 2.2-point decrease), we can regard the decrease in the mental health state at 2.5 years as corresponding to half of the magnitude of the decrease in physical health status during pregnancy. Thus, friends, relatives, and other contacts should provide more help to mothers because, in contrast to pregnant women, it is difficult to ascertain their situation from their appearance.

This study has several strengths. First, our sample size was large, including over 90,000 mothers. Second, the participants were enrolled from multiple regions throughout Japan. Third, we assessed various types of social support and trust. Fourth, the dropout rate was relatively low, at about 15.4%, despite follow-up to 2.5 years postpartum. Fifth, we included a wide range of covariates in the model. Finally, we used MSMs with IP weights to manage time-varying effects and censoring due to dropout.

This study also has some limitations. First, we included various covariates in the model but cannot deny the possible existence of unmeasured covariates, even though we conducted sensitivity analysis using *E*-values. Thus, the current effect estimates might be somewhat larger than the actual causal effects. Second, our participants were recruited between 2011 and 2014, so our data can be regarded as not fully reflecting recent situations in Japan. Although recent rapid developments in technology are changing how we communicate with others, we did not ask the participants which media they use for communication. One pertinent question is whether web conferences and/or chats have the same effects as face-to-face communication. Clearly, further studies examining this aspect are needed. Third, we measured social support in terms of emotional support but not other important aspects, such as instrumental, material, and informational support. Thus, further studies examining these aspects are necessary.

In conclusion, we found that a lack of social support and trust, particularly at only 2.5 years postpartum, has an adverse effect on the mental health of mothers but little effect on physical health. These findings suggest that a loss of social support and trust after childbirth carries high risk. Our results suggest the importance of providing continuous social support for mothers after childbirth, in addition to during pregnancy.

## SUPPLEMENTARY MATERIAL

eTable 1. Summary of the stabilized weights for each marginal structural model
