# Supplementary material for: Effect Estimate of Time-varying Social Support and Trust on the Physical and Mental Health of Mothers at 2.5 Years Postpartum: The Japan Environment and Children’s Study (JECS)
Source: J Epidemiol. 2023 Apr 5;33(4):177–85. doi: 10.2188/jea.JE20210134 (PMC9939921; doi:10.2188/jea.JE20210134)
Supplement: Supplementary file 1 [file je-33-177-s001.pdf]

eTable 1. Summary of the stabilized weights for each marginal structural model

Item	SW_A				SW_C				$SW_{A, C}$			
			Percentile				Percentile				Percentile	
	M	(SD)	1%	99%	M	(SD)	1%	99%	M	(SD)	1%	99%
Q1	1.00	0.16	0.66	1.58	1.00	0.10	0.90	1.39	1.00	0.20	0.70	1.72
Q2	1.00	0.16	0.82	1.35	1.00	0.10	0.90	1.39	1.00	0.19	0.83	1.65
Q3	1.00	0.20	0.63	1.52	1.00	0.10	0.90	1.39	1.00	0.22	0.65	1.74
Q4	1.00	0.19	0.92	1.31	1.00	0.10	0.90	1.39	1.00	0.22	0.87	1.65
Q5	1.00	0.25	0.49	1.91	1.00	0.10	0.89	1.39	1.00	0.28	0.53	2.00
Q6	1.00	0.25	0.50	1.91	1.00	0.10	0.89	1.39	1.00	0.27	0.54	2.00
Q7	1.01	0.18	0.59	1.74	1.00	0.10	0.90	1.39	1.01	0.22	0.63	1.87
Q8	1.01	0.25	0.47	1.94	1.00	0.10	0.89	1.39	1.01	0.27	0.50	2.02
Q9	1.01	0.31	0.62	1.63	1.00	0.10	0.90	1.39	1.01	0.31	0.64	1.90

M, mean; SD, standard deviation; SW, stabilized weight; SW_A, SW for treatment; SW_C, SW for censoring; $SW_{A, C}$, final SW.

Note: Q1–9 = item number on the 9-item questionnaire regarding social support and trust.
